# Is Fracture Toughness of PUR Foams a Material Property? A Statistical Approach

**DOI:** 10.3390/ma13214868

**Published:** 2020-10-30

**Authors:** Adrian Pugna, Romeo Negrea, Emanoil Linul, Liviu Marsavina

**Affiliations:** 1 Department of Management, University Politehnica Timisoara, Blvd. M. Viteazu, No. 1, 300222 Timisoara, Romania; adrian.pugna@upt.ro; 2 Department of Mathematics, University Politehnica Timisoara, Pta. Victoriei, No. 2, 300006 Timisoara, Romania; romeo.negrea@upt.ro; 3 Department of Mechanics and Strength of Materials, University Politehnica Timisoara, Blvd. M. Viteazu, No. 1, 300222 Timisoara, Romania; emanoil.linul@upt.ro

**Keywords:** polyurethane foam, fracture toughness, density, anisotropy, statistical approach

## Abstract

The published data on the experimentally determined fracture toughness of foams are based on a small number of specimens, having a lack of statistical consistency. The paper proposes a statistical approach on the fracture toughness results of rigid polyurethane (PUR) foams of three different densities. Five types of fracture tests were considered. The results were statistically analyzed using six types of regressions and a meta-analysis to identify the factors influencing the fracture toughness. The statistical analysis indicates that the fracture toughness represents a material property because does not depend on the specimen type. The density plays a major role in the fracture toughness of PUR foams. The irregular shape of the cells induced small anisotropy for low-density foams (100 kg/m^3^ and 145 kg/m^3^). This effect could not be observed for the foam with 300 kg/m^3^ density, for which the cells have a more regular spherical shape. The statistical analysis indicates that the influence of the loading speed is very weak.

## 1. Introduction

**Figure 1:**
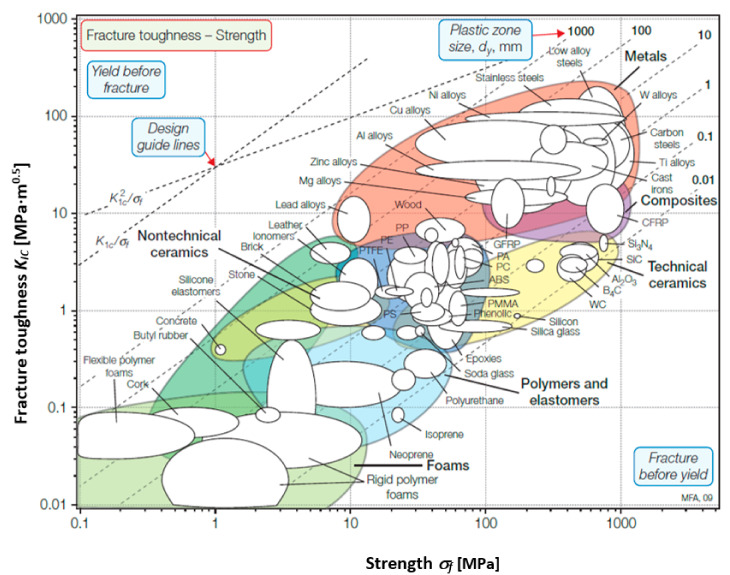
Fracture toughness versus strength [5].

For structural components, strength and fracture toughness are two important mechanical properties [1,2]. Yield strength is the measure of the stress that a material can withstand before plastic deformation, while the tensile strength is a measure of the maximum stress that a material can support before starting to fracture [3]. Fracture toughness is a measure of the energy required to fracture a material that contains a crack [4]. The relationship between fracture toughness and strength could be seen in a material selection diagram (Figure 1) [5].

It could be observed that foam materials are placed at the bottom left, corresponding with low fracture toughness (tenths of MPa·m^0.5^) and relatively low strength (up to 10 MPa).

In structural integrity applications, the fracture toughness represents a key material parameter, which plays an important role [6]. To measure fracture toughness of metals, extensive efforts have been made to develop reliable fracture toughness test methods since the 1960s. However, for polymeric materials, only the standard ASTM D5045–14 [7] describes the methodology to determine the plane-strain fracture toughness and strain energy release rate of plastic materials. Up to now, there are no standards for the determination of fracture toughness of cellular materials and plastic foams. However, often the methodology proposed in [7] was adopted for determination of fracture toughness of polymeric foams, considering Single Edge Notched Specimens loaded in Three-Point Bending [8,9,10], respectively Compact Tension specimens [11].

Jelitto and Schneider [12] revised the experimental methods and the fracture toughness data of porous materials including PUR foams, respectively Marsavina and Linul [13] presented a review of the fracture of polymeric foams.

**Figure 2:**
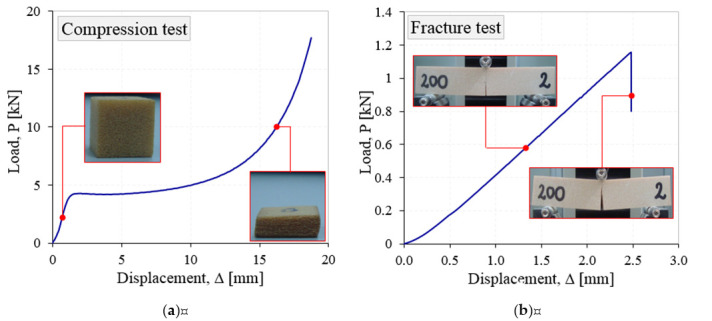
Load–displacement curves after compression (**a**) and three-point bending (**b**) tests.

It is also well known that the plastic foams have an elastic-plastic behavior in compression with a long plateau and a densification region Figure 2a [14,15], but a quasi-brittle behavior under tensile and in the presence of notches, cracks, Figure 2b, [16,17].

A limited number of works provide the fracture toughness of polyurethane (PUR) foams. Fowlkes [18] performed one of the first experimental investigation on fracture toughness of PUR foams with a density of 88 kg/m^3^, considering Middle Cracked, Double Edge Crack, Single Edge Crack and Double Cantilever Beam specimens. The fracture toughness was expressed by the critical energy release rate *G_IC_* and show that the fracture toughness results does not depend on specimen type. McIntyre and Anderton [8] presented the fracture toughness of PUR rigid foams in the density range 32–360 kg/m^3^. The tests were performed using Single Edge Notched Bend (SENB) specimens loaded in three-point bending. The obtained fracture toughness results range between 0.01 MPa·m^0.5^ for the lowest density foam, to 0.243 MPa·m^0.5^ for highest density foam. Kabir et al. [10] compared the fracture toughness of PUR and PVC foams with a density of 260 kg/m^3^, observing that the value of the fracture toughness for PVC is 2.2 times higher than the PUR one, and this is due to the higher toughness of the solid material from the cell walls.

Marsavina and Linul [19] experimentally investigated closed-cell rigid PUR foams with densities between 40 kg/m^3^ and 200 kg/m^3^ using SENB specimens loaded in three-point bending and determined the mode I fracture toughness between 0.034–0.39 MPa·m^0.5^. Fracture toughness of low-density rigid PUR (32–84 kg/m^3^) was obtained by Andersons et al. [11] in the range 0.022–0.058 MPa·m^0.5^ using Compact Tension (CT) specimens.

Poapongsakorn and Carlsson [20] using SENB specimens made of PVC foam showed that symmetric four point bending loading give a fracture toughness two times higher comparing with loading in three point bend configuration, due to indentation which occurs in the cracked cross section area, reducing the ligament size. 

Based on the experimental data, respectively on micromechanical modeling different authors expresses the fracture toughness of foams to the relative density of the foam (*ρ_f_/ρ_s_*), the dimension of the cell *l,* and tensile strength of the solid material, which forms the foam *σ_fs_* in the form:

(1) $$K_{IC}^{}=C_{}\sigma_{fs}\sqrt{\pi \;l}\;{\left(\frac{\rho_{f}}{\rho_{s}}\right)}^{m}[\mathrm{MPa}\cdot m^{0.5}]$$

with *C* a fitting constant, usually obtained by interrogating the experimental data.

Maiti et al. [21] proposed for the exponent *m* values of 1.5 for open cells, respectively 2 for closed-cell foams and for *C* a value of 0.65. Green [22] using an elastic model in shell theory of the hollow sphere found *C* = 0.28 and *m* = 1.3, while Choi and Sankar [23] taking into account the crack blunting and the non-singular stress field ahead of the crack tip proposed *C* = 0.19 and *m* = 1. 

However, all these models are determined usually on few experimental data having a lack of statistical consistency. In this regard, present paper proposes a statistical approach to the fracture toughness of rigid PUR foams. In the following section (Section 2), the investigated materials and experimental methodology are presented. Section 3 presents the influence of density, type of specimen, orientation and loading seed on the fracture toughness of PUR foams and is followed by the statistical assessment (Section 4). 

## 2. Materials and Methods 

**Figure 3:**
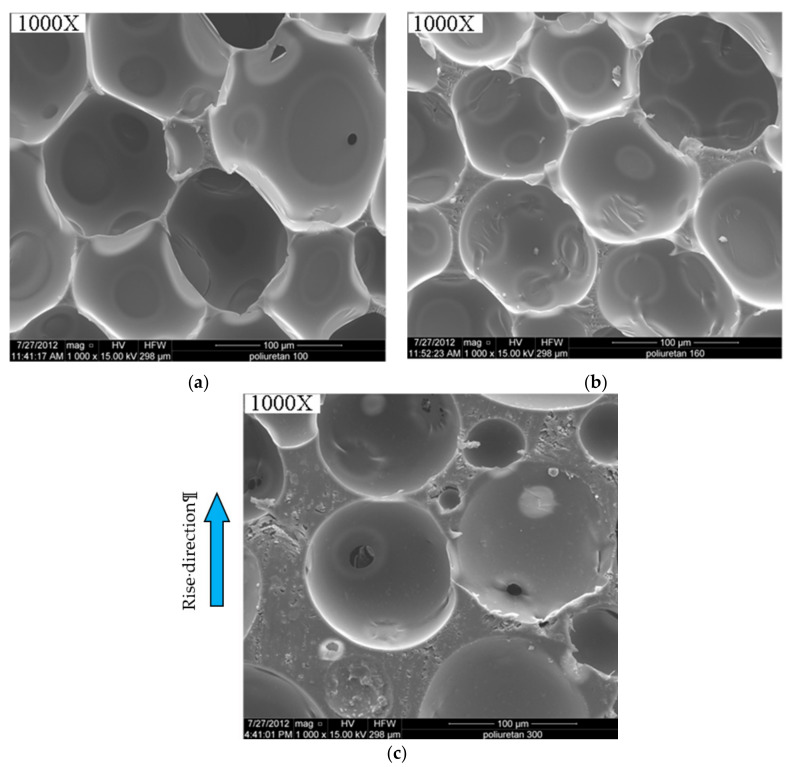
Microstructures of the investigated foams for 100 (**a**); 145 (**b**) and 300 (**c**) kg/m^3^.

Polyurethane (PUR) foams of three different densities (100 kg/m^3^, 145 kg/m^3^, and 300 kg/m^3^) were considered. The foams were produced by Necumer GbmH (Bohmte, Germany) under trade name NECURON 100, 160, and 301. Their microstructure is shown in Figure 3. The images were obtained with SEM QUANTATM FEG 250 (Hillsboro, OR, USA) at 1000× magnification.

**Figure 4:**
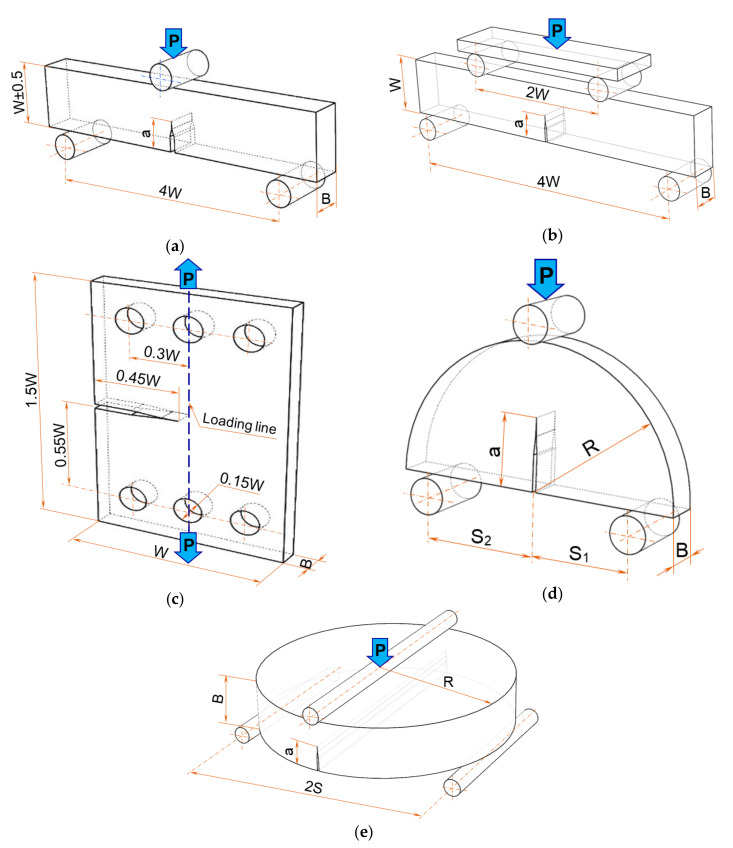
The geometry of the specimens and loading configuration of the fracture toughness tests: (**a**) Single Edge Notch Bend (SENB) specimen loaded in three point bending (*B* = 12.5 mm, *W* = 25 mm, *a* = 12.5 mm); (**b**) SENB specimen loaded in four point bending (*B* = 11.5 mm, *W* = 25 mm, *a* = 16.5 mm); (**c**) Single Edge Crack (SEC) specimen (*B* = 8 mm, *W* = 75 mm, *a* = 33.75 mm); (**d**) Symmetric Semi Circular Bend (SCB) specimen (*R* = 40 mm, *B* = 10 mm, *S*_1_ = *S*_2_ = 30 mm, *a* = 20 mm); (**e**) Edge Notch Bend Disc (ENDB) specimen (*R* = 75 mm, *B* = 30 mm, *a* = 15 mm).

The mode I fracture toughness tests were performed on different specimens: Single Edge Notch Bend (SENB) loaded in three (3PB) and four-point bending (4PB), Figure 4a,b, Single Edge Crack (SEC), Figure 4c, Semi-Circular Bend Specimen (SCB), Figure 4d, and Edge Notch Bend Disc (ENDB), Figure 4e. The specimens were cut in the flow and rise direction to study the foam anisotropy, but this was not possible for all types of specimens because the thickness of the foam plates was 50 mm for densities of 100 kg/m^3^ and 145 kg/m^3^, respectively 25 mm for the foam with 300 kg/m^3^ density.

Tests were carried out at room temperature with a loading speed of 2 and 50 mm/min using a ZWICK Z005 Proline (Ulm, Germany) universal testing machine.

The mode I fracture toughness values were determined with the maximum load *P_max_* from load–displacement curves, recorded during the experimental tests. Furthermore, the appropriate relationships, taking into account the geometry and dimensions of the specimens, were considered, Table 1.

The non-dimensional functions *f_I_(a/W)*, *g_I_(a/W)*, *h_I_(β, a/W)*, *j_I_(a/R, S_1_/R, S_2_/R)*, *k_I_(a/B, S/R, β)* are provided in the literature [7,24,25,26,27,28].

## 3. Experimental Results

The fracture toughness results for the three investigated foams are presented in Table 2, Table 3 and Table 4. It could be observed that the average values for the 100 kg/m^3^ density PUR foam were between 0.071–0.091 MPa·m^0.5^ in the flow direction, respectively up to 0.106 MPa·m^0.5^ in the rise direction. 

The foam with 145 kg/m^3^ density has an average value of the fracture toughness in the flow direction in the range 0.095–0.132 MPa·m^0.5^, respectively for the rise direction between 0.116–0.143 MPa·m^0.5^.

Finally, for the foam with a density of 300 kg/m^3^, the fracture toughness values were obtained between 0.337–0.372 MPa·m^0.5^ in the flow direction, respectively 0.344 MPa·m^0.5^ in the rise direction. 

Overall, it could be pointed out that the fracture toughness increases with density [29].

It could be observed that the anisotropy effect is higher for low-density foams (100 kg/m^3^ and 145 kg/m^3^) and diminished for 300 kg/m^3^ density. This could be explained based on the cell topology (cells have different shapes in flow and rise direction), while for the foam with 300 kg/m^3^ density cells are more regular like spheres [30].

## 4. Discussion: A Statistical Approach

The statistical analysis aimed at two objectives. The first is to determine the relationships between the fracture toughness of foams and different variables measured in several experiments. For this purpose, the regression method will be used [31]. The second goal is to analyze if the fracture toughness depends on the different densities and different types of specimens. This problem is equivalent to the problem of determining the effect size. For the second objective, the meta-analysis method will be considered [32].

For the statistical analysis it is denoted Y for the response variable, the mean value of the fracture toughness and five predictor variable: X1 - specimen type (1 = ”SENB-TPB”, 2 = ”SENB-FPB”, 3 = ”SEC”, 4 = ”ASCB”, 5 = ”ENDB”), X2—loading speed (2 or 50), X3 - density (100 kg/m^3^, 145 kg/m^3^ or 300 kg/m^3^), X4 = direction plane (1 = ”in-plane-flow”, 0 = ”out of plane-rise”), X5 = number of measurements (2, 3, 4, 5). 

The correlation matrix (see Table 5) shows some possible linear relations between the response and the predictors, also some relations between the predictors are given. Each cell of this matrix represents the Pearson correlation coefficient between any two of the variables X1, X2, X3, X4, X5, or Y describes above.

The multiple linear regression method and the backward selection procedure were used. The optimization process led to the conclusion that the response variable fracture toughness depends only on one predictor variable (density). Different regression models were analyzed. The goal of the regression analysis was to find the best relation between the response variable Y (mean value of the fracture toughness) and the predictor X3 (density). There exists some studies that indicated a linear relation (Y = a + b × X3), a quadratic relation (Y = a + c × X3^2^) or a power relation (Y = a × X^1.3^, see [22]). In our analysis, we consider these models and other related models as follows:

1. Linear model, Y = a + b × X3 (Table 6).

Results: The coefficient of determination R^2^ = 0.9789, adjusted coefficient of determination Ra^2^ = 0.9696. The model is statistically supported, with an error of less than 1% and a coefficient of determination of over 96% (below are the statistical indicators, using the R software (R version 4.0.0).

The above table presents:

- Column Estimate—the estimation values of the parameters (estimations using least square method).
- Column Std. Erros—the statistical standard deviation of the parameters.
- Column t-value—the value of t test, which it is used to verify the null hypothesis H_0_: a = 0, respectively H_0_: b = 0, the expression of this test is estimated value / standard deviation.
- Column Pr(>|t|)—the probability to not reject the null hypothesis. It is obvious that some null value of the parameters (accept the null hypothesis) are not desirable because this means that the predictor is not significant, so a value small as possible is good to accept the proposed model.
- Residual standard error—the sum of values between the observed (measured) value and the value predicted by model (it is desirable to be small as possible), degrees of freedom is the number of observations minus the number of parameters.
- Multiple R-squared—the coefficient of determination 1-(Residual sum of squares/Total sum of squares), (Residual sum of squares is the sum of square distance between the observed values and the values predicted by model, total sum of squares is the sum of square distance between the observed values and the statistical (arithmetical) mean of observations), it is obvious that this value should be large as possible;
- Adjusted R-squared is a correction expression of the coefficient of determination (because this coefficient tends to increase with increasing number of parameters (i.e., predictors).
- F-statistic—the value of F-test to verify the null hypothesis H_0_ a = b = 0 (both parameters are zero).
- *p*-value is the probability to not reject the null hypothesis, of course, a low value as possible it useful to accept the model.

2. Quadratic Model, Y = a + c × X3^2^ (Table 7)

Coefficient of determination R^2^ = 0.9847, adjusted coefficient of determination Ra^2^ = 0.9839.

3. Polynomial (second-order) model Y = b·X3 + c·X3^2^ (Table 8)

Coefficient of determination R^2^ = 0.9943, adjusted coefficient of determination Ra^2^ = 0.9938.

4. Power model Y = a·X3^b^ (Table 9)

Coefficient of determination R^2^ = 0.9421, adjusted coefficient of determination R_a_^2^ = 0.9393.

This model can be obtained using a “linearization” method, applying the logarithm function the power model become a “linear” model. The linearization method does not lead the best estimates of parameters, but it gives an “initial” solution for the nonlinear least square method. 

From above, we observe that a good model seems to be by the form Y = a·X3^b^. We use a nonlinear regression procedure to estimate the parameters “a” and “b” (see Table 10). 

In a nonlinear regression can be computed just a quasi-coefficient of determination using the same relation as that described after the Table 6. To compare more nonlinear regression models can be used some informational criterion as AIC (Akaike’s Information Criterion, 2 * number of parameters—logarithm of the likelihood function) or BIC (Bayesian Informational Criterion). Also, we can mention that for the “initial” values b = 1 or b = 2, the nonlinear least square method yields the same value b = 1.366. 

Using the above estimation (b = 1.366) we consider the following power model (5):

5. Power model Y = a + b·X3^1.366^ (Table 11)

Coefficient of determination R^2^ = 0.9793, adjusted coefficient of determination R_a_^2^ = 0.9783.

Also, these values are obtained using the linearization method. 

Because the *p*-value of the free term (intercept) is too big (0.69) we will analyze the above model without intercept (6):

6. Power model Y = b·X3^1.366^ (Table 12)

Coefficient of determination R^2^ = 0.9937, adjusted coefficient of determination R_a_^2^ = 0.9934. Again, we used the linearization method to calculate these values. 

Statistically, the best models are the polynomial model (model d) and the last power model (model h) (because explains 99 % of situations). All models are made with an error of less than 1%. (chosen significance level alpha = 0.01). Because are just some very small differences between the values of coefficient of determination at these models can be sustain that are equally good statistically. Also, follows the remark that for nonlinear model we obtained a value of this coefficient based on linearization method, the nonlinear model seems to be better. 

A meta-analysis was performed to study possible measurement errors (Table 13). The values for density = 100 kg/m^3^ were considered as the control group and the values for density = 145 kg/m^3^ as the experimental group. Data obtained using the R software and the meta-package [33]. The fixed effect model provides a weighted average of a series of study estimates. A common model used to synthesize heterogeneous research is the random effects model of meta-analysis. For details of the values from below table, see [32,33].

It is observed that there is a difference between the fixed and the random effect, in other words, the difference between the average values depends on a fixed effect (density difference) and there are only small differences due to chance (normal statistical errors).

In addition, a meta-analysis was performed to study whether there was an effect on the difference between the types of specimens (Table 14). The SEC specimen was considered as a control model and the ENDB specimen as an experimental model.

It is observed that the fixed effect coincides with the random one, in other words, the difference is only due to chance and it cannot be stated that there is a difference due to the choice of a certain type of specimen.

## 5. Conclusions

The statistical analysis based on the experimental results indicates that the fracture toughness represents a material property of PUR foams, because does not depend on the specimen type. From the practical point of view, this conclusion is important, allowing to consider any type of specimen, based on the availability, to determine the fracture toughness. On other hand, the fracture criterion based on the stress intensity factor *K_I_* and on the critical value, also known as fracture toughness *K_IC_*: *K_I_* ≤ *K_IC_*, could be successfully applied to structures made of PUR foams. The size effect shows that for large PUR foams structures the linear elastic fracture mechanics applies [13,27] and the fracture parameters have a crucial role on the integrity assessment. 

The density plays a major role in the fracture toughness of PUR foams. The statistical analysis based on the experimental data shows that the linear model, the power models, and the square root model fitted the micromechanical models described by eq. (1) with resulting values for *m* = 1, 1.366, 1.5, all having a coefficient of determination higher than 0.94. The highest being obtained for the power model with *m* = 1.366. Also, a combined model between model with *m* = 1 and model with *m* = 2 seems to be good. These results based on the statistical analysis are in agreement with other published data [21,22,23].

The irregular shape of the cells induced small anisotropy for low-density foams (100 kg/m^3^ and 145 kg/m^3^). This effect could not be observed for the foam with 300 kg/m^3^ density, for which the cells have a more regular spherical shape. 

The statistical analysis indicates that the influence of the loading speed is very weak.

## Figures and Tables

**Table 1:** Fracture toughness calculation.

Specimen Type	Calculation of K_IC_	Reference
SENB loaded in 3PB	$K_{IC}=\frac{3P_{\max}S}{2B_{}W^{2}}\;f_{I}\left(a/W\right)$	[7]
SENB loaded in 4PB	$K_{IC}=\frac{3P_{\max}}{B_{}W}\;g_{I}\left(a/W\right)$	[24,25]
SEC	$K_{IC}=\frac{p_{\max}}{W\;t}\sqrt{\pi a}\;h_{I}\left(\beta \;,\;a/W\right)\;$	[26]
SCB	$K_{IC}=\frac{P_{\max}}{2Rt}\sqrt{\pi a}\;j_{I}\left(a/R,\;S_{1}/R,\;S_{2}/R\right)\;$	[27]
ENBD	$K_{IC}=\frac{6P_{\max}S}{R_{}B^{2}}\;k_{I}\left(a/B,S/R,\beta \right)$	[28]

**Table 2:** Fracture toughness results for 100 kg/m^3^ polyurethane (PUR) foam density.

Specimen Type	Loading Direction	Loading Speed [mm/min]	Fracture Toughness [MPa·m^0.5^]					
			1	2	3	4	5	Average
SENB-TPB	Flow	2	0.072	0.074	0.075	0.068	–	0.072
SENB-TPB	Rise	2	0.070	0.082	0.078	0.076	–	0.076
SENB-FPB	Flow	2	0.072	0.071	0.070	0.073	0.071	0.071
SEC	Flow	2	0.083	0.090	0.079	0.100	–	0.088
SCB	Flow	2	0.084	0.088	0.090	–	–	0.087
SCB	Flow	50	0.074	0.105	0.095	–	–	0.091
SCB	Rise	2	0.109	0.099	0.116	0.102	–	0.106
SCB	Rise	50	0.089	0.095	–	–	–	0.092
ENDB	Rise	2	0.094	0.093	0.087	–	–	0.091

**Table 3:** Fracture toughness results for 145 kg/m^3^ PUR foam density.

Specimen Type	Loading Direction	Loading Speed [mm/min]	Fracture Toughness [MPa·m^0.5^]						
			1	2	3	4	5	6	Average
SENB-TPB	Flow	2	0.102	0.105	0.099	0.107	0.119	0.124	0.109
SENB-TPB	Rise	2	0.110	0.111	0.109	0.111	0.128	0.125	0.116
SENB-FPB	Flow	2	0.091	0.092	0.099	0.093	0.102	–	0.095
SEC	Flow	2	0.124	0.100	0.128	0.106	0.084	–	0.109
SCB	Flow	2	0.135	0.134	0.129	0.129	–	–	0.132
SCB	Flow	50	0.128	0.136	–	–	–	–	0.132
SCB	Rise	2	0.139	0.138	0.151	0.145	–	–	0.143
SCB	Rise	50	0.128	0.136	–	–	–	–	0.132
ENDB	Rise	2	0.108	0.117	0.113	–	–	–	0.113

**Table 4:** Fracture toughness results for 300 kg/m^3^ PUR foam density.

Specimen Type	Loading Direction	Loading Speed [mm/min]	Fracture Toughness [MPa·m^0.5^]					
			1	2	3	4	5	Average
SENB-TPB	Flow	2	0.325	0.343	0.373	0.330	0.327	0.340
SENB-FPB	Flow	2	0.362	0.362	0.321	0.345	0.349	0.348
SEC	Flow	2	0.432	0.284	0.320	0.311	–	0.337
SCB	Flow	2	0.356	0.384	0.377	–	–	0.372
ENDB	Rise	2	0.343	0.347	0.342	–	–	0.344

**Table 5:** Correlation matrix.

Variable	X1	X2	X3	X4	X5	Y
X1	1.00000000	0.3034885	−0.03203788	−0.3367175	−0.7642309	0.0396120
X2	0.30348849	1.0000000	−0.23335505	−0.1021899	−0.6698430	−0.1988581
X3	−0.03203788	−0.2333551	1.00000000	0.1993570	0.1841807	0.9853670
X4	−0.33671751	−0.1021899	0.19935697	1.0000000	0.2661637	0.1681640
X5	−0.76423093	−0.6698430	0.18418069	0.2661637	1.0000000	0.1289626
Y	0.03961200	−0.1988581	0.98536705	0.1681640	0.1289626	1.0000000

**Table 6:** Statistical parameters for the linear regression model.

	Estimate	Std. Error	t Value	Pr (>|t|)
a	−5.895e−02	0.984e−03	−6.561	1.69e−06 ***
b	1.337e−03	5.045e−05	26.492	<2e−16 ***
Residual Standard Error: 0.01836 on 21 Degrees of Freedom				
Multiple R-Squared: 0.9709, Adjusted R-Squared: 0.9696				
F-Statistic: 701.8 on 1 and 21 DF, *p*-Value: < 2.2e−16				

*** The value is less than 0.001.

**Table 7:** Statistical parameters for the quadratic regression model.

	Estimate	Std. Error	t Value	Pr (>|t|)
a	5.229e−02	3.70e−03	13.17	1.29e−11 ***
c	3.283e−06	8.939e−08	36.73	<2e−16 ***
Residual Standard Error: 0.01334 on 21 Degrees of Freedom				
Multiple R-Squared: 0.9847, Adjusted R-Squared: 0.9839				
F-Statistic: 1349 on 1 and 21 DF, *p*-Value: < 2.2e−16				

*** The value is less than 0.001.

**Table 8:** Statistical parameters for the polynomial regression model.

	Estimate	Std. Error	t Value	Pr (>|t|)
b	6.066e−04	5.165e−05	11.744	1.08e−10 ***
c	1.831e−06	2.071e−07	8.845	1.59e−08 ***
Residual Standard Error: 0.01475 on 21 Degrees of Freedom				
Multiple R-Squared: 0.9943, Adjusted R-Squared: 0.9938				
F-Statistic: 1842 on 1 and 21 DF, *p*-Value: < 2.2e−16				

*** The value is less than 0.001.

**Table 9:** Statistical parameters for the power regression model.

	Estimate	Std. Error	t Value	Pr (>|t|)
a	−8.41281	0.34679	−24.26	<2e−16 ***
b	1.28048	0.06927	18.48	1.8e−14 ***
Residual standard error: 0.1363 on 21 Degrees of Freedom				
Multiple R-Squared: 0.9421, Adjusted R-Squared: 0.9393				
F-Statistic: 341,7 on 1 and 21 DF, *p*-Value: 1.801e−14				

*** The value is less than 0.001.

**Table 10:** Statistical parameters for the nonlinear regression model.

	Estimate	Std. Error	t Value	Pr (>|t|)
a	1.430e−04	3.721e−05	3.844	0.000943 ***
b	1.366e + 00	4.723e−02	28.924	<2e−16 ***
Residual Standard Error: 0.01565 on 21 Degrees of Freedom				
Number of Iterations to Convergence: 7				
Achieved Convergence Tolerance: 5.974e−07				

*** The value is less than 0.001.

**Table 11:** Statistical parameters for the power regression model Y = a + b·X3^1.366^.

	Estimate	Std. Error	t Value	Pr (>|t|)
a	2.370e−13	5.853e−03	0.405	0.69
b	1.416e−04	4.488e−06	31.544	<2e−16 ***
Residual Standard Error: 0.01549 on 21 Degrees of Freedom				
Multiple R-Squared: 0.9793, Adjusted R-Squared: 0.9783				
F-Statistic: 995 on 1 and 21 DF, *p*-Value: < 2.2e−16				

*** The value is less than 0.001.

**Table 12:** Statistical parameters for the power regression model Y = b·X3^1.366^.

	Estimate	Std. Error	t Value	Pr (>|t|)
B	1.431e−04	2.428e−06	58.93	<2e−15 ***
Residual Standard Error: 0.01519 on 21 Degrees of Freedom				
Multiple R-Squared: 0.9937, Adjusted R-Squared: 0.9934				
F-Statistic: 3472 on 1 and 21 DF, *p*-Value: < 2.2e−16				

*** The value is less than 0.001.

**Table 13:** Meta-analysis of densities.

Number of Studies Combined: k = 9				
	**SMD**	**95%-CI**	**z**	***p*-Value**
Fixed Effect Model	3.0705	[1.9954; 4.1455]	5.60	<0.0001
Random Effects Model	3.7582	[2.1870; 5.3294]	4.69	<0.0001
Quantifying Heterogeneity:				
tau^2^ = 1.9157; H = 1.27 [1.00; 1.87]; I^2^ = 37.5% [0.0%; 71.3%]				
Test of Heterogeneity:				
	**Q**	**DF**	***p*-Value**	
	12.80	8	0.1187	
Details on Meta-Analytical Method:				
Inverse Variance Method DerSimonian-Laird Estimator for tau^2^ Hedges’ g (bias-Corrected Standardized Mean Difference)				

**Table 14:** A meta-analysis of specimen types.

Number of Studies Combined: k = 3				
	**SMD**	**95%-CI**	**z**	***p*-Value**
Fixed Effect Model	0.2373	[−0.6350; 1.1095]	0.53	0.5940
Random Effects Model	0.2373	[−0.6350; 1.1095]	0.53	0.5940
Quantifying Heterogeneity:				
tau^2^ = 0; H = 1.00 [1.00; 1.00]; I^2^ = 0.0% [0.0%; 0.0%]				
Test of Heterogeneity:				
	**Q**	**DF**	***p*-Value**	
	0.05	2	0.9743	
Details on Meta-Analytical Method:				
Inverse Variance Method DerSimonian-Laird Estimator for tau^2^ Hedges’ g (bias-Corrected Standardized Mean Difference)				
